# 新型样品前处理材料在环境污染物分析检测中的研究进展

**DOI:** 10.3724/SP.J.1123.2021.02030

**Published:** 2021-08-08

**Authors:** Juanjuan FENG, Xiangping JI, Chunying LI, Mingxia SUN, Sen HAN, Jiaqing FENG, Haili SUN, Yang FENG, Min SUN

**Affiliations:** 济南大学化学化工学院, 山东 济南 250022; School of Chemistry and Chemical Engineering, University of Jinan, Jinan 250022, China; 济南大学化学化工学院, 山东 济南 250022; School of Chemistry and Chemical Engineering, University of Jinan, Jinan 250022, China; 济南大学化学化工学院, 山东 济南 250022; School of Chemistry and Chemical Engineering, University of Jinan, Jinan 250022, China; 济南大学化学化工学院, 山东 济南 250022; School of Chemistry and Chemical Engineering, University of Jinan, Jinan 250022, China; 济南大学化学化工学院, 山东 济南 250022; School of Chemistry and Chemical Engineering, University of Jinan, Jinan 250022, China; 济南大学化学化工学院, 山东 济南 250022; School of Chemistry and Chemical Engineering, University of Jinan, Jinan 250022, China; 济南大学化学化工学院, 山东 济南 250022; School of Chemistry and Chemical Engineering, University of Jinan, Jinan 250022, China; 济南大学化学化工学院, 山东 济南 250022; School of Chemistry and Chemical Engineering, University of Jinan, Jinan 250022, China; 济南大学化学化工学院, 山东 济南 250022; School of Chemistry and Chemical Engineering, University of Jinan, Jinan 250022, China

**Keywords:** 样品前处理, 金属有机框架, 共价有机框架, 分子印迹材料, 碳纳米管, 气凝胶, 三嗪基材料, 环境污染物, sample pretreatment, metal-organic frameworks, covalent organic frameworks, molecular imprinting materials, carbon nanotubes, aerogels, triazine-based materials, environmental pollutants

## Abstract

针对复杂样品的分析和痕量目标物的检测,样品前处理是必不可少的,高效的样品前处理技术不仅可以去除或减小样品基质干扰而且能够实现分析物的富集,提高分析检测的准确性和灵敏度。近年来,固相萃取、磁分散固相萃取、枪头固相萃取、搅拌棒萃取、固相微萃取等高效的样品前处理技术已在环境污染物分析检测中获得广泛关注,萃取效率主要取决于萃取材料,所以新型的高效萃取材料一直是样品前处理研究领域的重要发展方向。该文总结和讨论了近年来新型样品前处理材料在环境污染物分析检测中的研究进展,主要聚焦在石墨烯、氧化石墨烯、碳纳米管、无机气凝胶、有机气凝胶、三嗪基功能材料、三嗪基聚合物、分子印迹聚合物、共价有机框架材料、金属有机框架材料以及它们的功能化萃取材料等。这些材料已经被应用于环境样品中不同类别污染物的萃取富集,如重金属离子、多环芳烃、塑化剂、烷烃、苯酚、氯酚、氯苯、多溴联苯醚、全氟磺酸、全氟羧酸、雌激素、药物残留、农药残留等。这些样品前处理材料具有高的表面积、大量的吸附位点,并涉及多种萃取机理如*π-π*、静电、疏水、亲水、氢键、卤键等相互作用。基于这些萃取材料的多种样品前处理技术与各类检测方法如色谱、质谱、原子吸收光谱、荧光光谱、离子迁移谱等相结合,已广泛应用于环境污染物的高灵敏分析检测。最后,该文总结了样品前处理发展中存在的问题,并展望了其未来在环境分析中的发展趋势。

## Abstract (en)

To successfully analyze complex samples and detect trace targets, sample pretreatment is essential. Efficient sample pretreatment techniques can remove or reduce interference from the sample matrix. It can also enrich analytes, thereby improving analytical accuracy and sensitivity. In recent years, various sample preparation techniques, including SPE, magnetic dispersion SPE, pipette tip SPE, stir bar extraction, fiber SPME, and in-tube SPME, have received increasing attention in environmental analysis and monitoring. The extraction efficiency mainly depends on the type of adsorbent material. Therefore, the development of efficient adsorbents is a crucial step toward sample preparation. This review summarizes and discusses the research advances in extraction materials over recent years. These extraction materials contain inorganic adsorbents, organic adsorbents, and inorganic-organic hybrid materials such as graphene, graphene oxide, carbon nanotubes, inorganic aerogels, organic aerogels, triazinyl-functionalized materials, triazine-based polymers, molecularly imprinted polymers, covalent organic frameworks, metal-organic frameworks, and their derivatives. These materials have been applied to extract different types of pollutants, including metal ions, polycyclic aromatic hydrocarbons, plasticizers, alkanes, phenols, chlorophenols, chlorobenzenes, polybrominated diphenyl ethers, perfluorosulfonic acids, perfluorocarboxylic acids, estrogens, drug residues, and pesticide residues, from environmental samples (such as water and soil samples). These sample preparation materials possess high surface areas, numerous adsorption sites, and allow extraction via various mechanisms, such as *π-π*, electrostatic, hydrophobic, and hydrophilic interactions, as well as hydrogen and halogen bond formation. Various sample pretreatment techniques based on these extraction materials have been combined with various detection methods, including chromatography, mass spectrometry, atomic absorption spectroscopy, fluorescence spectroscopy, and ion mobility spectroscopy, and have been extensively used for the determination of environmental pollutants. The existing challenges associated with the development of sample preparation techniques are proposed, and prospects for such extraction materials in environmental analysis and monitoring are discussed. Major trends in the field, including the development of efficient extraction materials with high enrichment ability, good selectivity, excellent thermal stability, and chemical stability, are discussed. Green sample pretreatment materials, environmentally friendly synthesis methods, and green sample pretreatment methods are also explored. Rapid sample pretreatment methods that can be conducted within minutes or seconds are of significant interest. Further, online sample pretreatment and automatic analysis methods have attracted increasing attention. Besides, real-time analysis and in situ detection have been important development directions, and are expected to be widely applicable in environmental analysis, biological detection, and other fields. Modern synthesis technology should be introduced to synthesize specific extraction materials. Controllable preparation methods for extraction materials, such as the in situ growth or in situ preparation of extraction coatings, will acquire importance in coming years. It will also be important to adopt high-performance materials from other fields for sample pretreatment. Organic-inorganic hybrid extraction materials can combine the advantages both organic materials and inorganic materials, and mutually compensate for any disadvantages. Extraction materials doped with nanomaterials are also promising. Although existing sample pretreatment techniques are relatively efficient, it is still imperative to develop novel sample preparation methods.

近年来环境污染越来越受到人们的重视,对环境中存在的污染物进行准确、高灵敏分析检测至关重要。但是部分污染物的含量低,且环境样品基质复杂,限制了环境监测的过程和结果的准确性,因此需要高效的样品前处理技术,才能实现目标分析物的检测。而传统样品前处理方法,如液液萃取、索式提取、蒸馏、离心、过滤等,存在有机溶剂消耗量大、费时费力、富集效率低、重现性差等问题^[1]^。近几十年来,样品前处理技术获得了飞速发展,为了克服上述问题,出现了柱固相萃取(CSPE)、分散固相萃取(DSPE)、磁分散固相萃取(MSPE)、移液枪头固相萃取(PTSPE)、纤维固相微萃取(fiber SPME)、管内固相微萃取(IT-SPME)、中空纤维萃取(hollow fiber extraction)、搅拌棒萃取(stir bar extraction)等多种新技术。这些萃取方法样品用量少,抗基体干扰强,有机溶剂消耗量低(甚至无溶剂萃取),富集效率高,萃取时间短,操作简便,便于与色谱分析技术实现在线或半自动联用^[2]^。这些新型样品前处理技术都是基于吸附剂的萃取方法,吸附材料的性能直接制约着这些方法的萃取行为,所以新型高性能萃取材料的制备和应用一直是近年来的研究热点。

近年来,各种微米、纳米材料层出不穷,有力推动了新型样品前处理材料的发展。无机材料具有良好的机械强度、优异的热稳定性和化学稳定性,在样品前处理材料中有良好的应用前景。碳纳米材料如石墨烯(G)、氧化石墨烯(GO)和碳纳米管(CNTs)被引入SPE、SPME等技术中,应用于环境污染物的检测^[3]^。气凝胶材料作为已知世界上最轻的多孔材料,其比表面积大、孔隙度高,已从隔热、吸附等领域逐渐应用到样品前处理中^[4]^。最早出现的气凝胶材料是无机气凝胶,后来又发展了有机气凝胶。有机材料具有易于设计和调控化学结构的独特性质,在样品前处理领域中也一直备受关注。分子印迹材料就是一类代表性的有机材料,通过印迹位点能够实现特异性识别目标分子,在样品前处理领域中发挥着不可替代的作用^[5]^。为了改善无机材料的吸附性能,可以利用有机官能团对其进行功能化,如三嗪基团可以与各类分析物产生多种相互作用机理,使得三嗪基材料在环境样品的预处理中表现出良好的萃取性能。在近年来发展的新型先进材料中,共价有机框架材料(COFs)、金属有机框架材料(MOFs)吸引了人们的广泛关注,并在多个研究领域表现出良好的潜力,在样品前处理研究领域也获得了较多应用^[6]^。本论文先从典型的无机碳纳米材料石墨烯、碳纳米管开始,再讨论无机气凝胶,并过渡到有机材料中的有机气凝胶,进而综述三嗪基材料,再总结共价有机框架材料和分子印迹材料两大类重要的有机样品前处理材料,最后描述金属有机框架材料这一类重要的无机-有机杂化材料在环境样品前处理中的应用。如图1所示,本论文结合自己课题组的相关研究工作对上述新型萃取材料在环境污染物分析检测中的研究进展进行了详细综述。

**图1:**
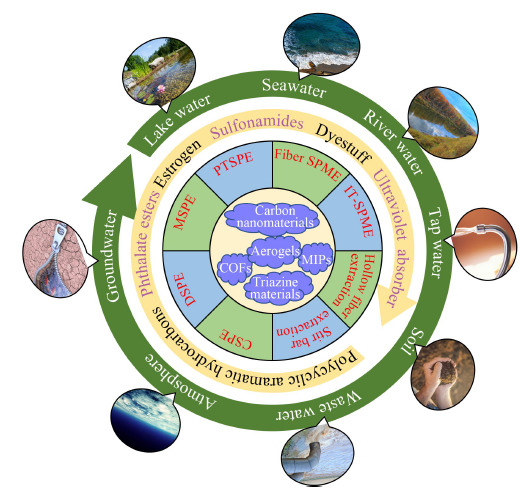
新型样品前处理材料在环境污染物分析检测中的研究进展

## 1 碳纳米材料

纳米材料凭借超高比表面积、优异的吸附性能、易修饰性和可调节的光学性质等独特优点,已被广泛应用于储能、催化、分离富集、药物释放和环境修复等领域^[7]^。常见碳纳米材料包括G、GO、单壁碳纳米管(SWCNTs)、多壁碳纳米管(MWCNTs)、碳量子点、碳纳米纤维等,它们兼具碳材料(优异的物理和化学特性、机械或电子性质)和纳米材料(大比表面积、高孔结构和纳米尺度)的特性,已被广泛用作样品前处理材料。它们可以提供多种相互作用来吸附分析物,以获得满意的萃取能力^[8]^。但是,碳纳米材料对分析物的萃取选择性差,尤其是复杂基体样品中存在多类别分析物时,通过-COOH^[9]^、$-NH_{2}$^[10]^等官能团对碳纳米材料进行改性,或是借助其他纳米材料^[11]^、离子液体(ILs)^[12]^、聚合物^[13]^等对其进行功能化,能够有效改善其萃取选择性。

### 1.1 石墨烯

作为一种*sp*^2^键合碳原子单层厚度呈蜂窝状排列的二维(2D)结构,G表现出非凡的优点,如具有比表面积大(理论值2630 m^2^/g)、双面多环芳烃骨架、固有的分子吸附两面性、易于表面修饰和大的*π*电子体系。G被认为是一种高性能的吸附剂,特别是对于芳香化合物^[14,15]^。然而,由于其自身的非极性和强疏水性,这导致G在水相中易聚集,难分散,阻碍了对分析物的有效吸附和解析^[16]^。 相比之下,GO作为一种层状的含氧G片,在其边缘和表面上具有大量的极性官能团(-OH、-CHOCH-、-C=O、-COOH等),因此GO比G更具亲水性和极性,能在水溶液中形成稳定的胶体悬浮液,提高了对极性化合物的吸附亲和力。此外,当pH>3.9时,GO的表面带负电,这意味着GO在一定pH范围内具有吸附阳离子的能力^[17]^。近年来,G和GO基材料被广泛用作SPE和SPME吸附剂,从环境水样中萃取痕量目标分析物(例如重金属离子、多环芳烃、雌激素、杀菌剂、除草剂和农药等),相关具体应用总结在表1中。

**表1:** 石墨烯和氧化石墨烯基样品前处理材料在环境污染物分析检测中的应用

Adsorbent	Analytes	Sample	LODs/(μg/L)		Linear range/(μg/L)			Analytical method
GO-PDAP^[18]^	Cd^2+^	water	0.47		2	-100	SPE-FAAS	
GO/polyaniline^[19]^	Cd^2+^	water	0.1		0.4	-1000	SPE-DLLME-FAAS	
Al_2_O_3_/GO^[20]^	Cr^3+^, As^5+^	water	0.11	, 0.02	2.0	-50	D-μ-SPE-EDXRF	
POT/GO^[21]^	three nonsteroidal anti- inflammatory drugs	water	0.02	-0.03	0.08	-200	D-μ-SPE-HPLC-UV	
GO@NH_2_@Fe_3_	twelve quinolones	water	10.0			-	MSPE-MALDI-TOF MS	
M-MOF-199^[22]^	five triazole pesticides	water	0.05	-0.1	0.25	-1000	MSPE-HPLC-MS/MS	
Fe_3_O_4_@HP-β-CD-RGO^[23]^	Cd^2+^	water	0.23		0.50	-100.0	MSPE-FAAS	
MG/PDA^[24]^	four benzoylurea insec- ticides	water	0.75		2.5	-500	MDSPE-HPLC-DAD	
MG/CNTs/PDA^[25]^	sixteen PAHs	water	0.0001	-0.003	0.010	-0.500	MSPE-GC-MS	
MGO@mSiO_2_-MIPs^[26]^	six PAEs	water	0.01	-0.05	1	-50	MSPE-GC-MS	
GO@Fe_3_O_4_-MIP^[27]^	microsystin-LR	water	0.08		2	-10000	MSPE-HPLC-UV	
G^[28]^	six PAHs	water	0.01	-0.09	0.05	-50	HS-SPME-GC-FID	
3D-rGO-PANI^[29]^	ethion	water	0.4		1.0	-70	DI-SPME-HPLC	
GO^[30]^	five PAHs	water	0.05	-0.10	0.5	-200	SPME-GC	
G^[31]^	five n-alkanes	water	0.05	-0.50	0.2	-150	SPME-GC	
GO reinforced PILs monolith^[32]^	phenols	water	0.20	-0.50	5	-400	SPME-HPLC	
GO-CFs^[33]^	ten PAHs	wastewater	0.001	-0.004	0.003	-50	IT-SPME-HPLC-DAD	

PDAP: poly(2,6-diaminopyridine) composite; POT/GO: polyoxotungstate anion functionalized GO; GO@NH_2_@Fe_3_O_4_: GO-functionalized magnetic composites; M-MOF-199: magnetic copper metal-organic framework-199; Fe_3_O_4_@HP-*β*-CD-RGO: hydroxypropyl-*β*-cyclodextrin functionalized reduced graphene oxide grafted on magnetic Fe_3_O_4_ microspheres; MG/PDA: magnetic graphene/polydopamine; MG/CNTs/PDA: polydopamine functionalized magnetic G and CNTs hybrid nanocomposites; MGO@mSiO_2_-MIPs: molecularly imprinted polymers coated magnetic graphene oxide with mesoporous silica; GO@Fe_3_O_4_-MIP: Fe_3_O_4_@GO-molecular imprinting polymers; 3D-rGO-PANI: three-dimentional reduced graphene oxide-polyaniline composite; CFs: carbon fibers; PAHs: polycyclic aromatic hydrocarbons; PAEs: phthalates; -: no data; FAAS: flame atomic absorption spectrometry; DLLME: dispersive liquid-liquid microextraction; D-μ-SPE: dispersive micro-SPE; EDXRF: energy-dispersive fluorescence spectrometry; MSPE: magnetic solid phase extraction; HS: headspace; DI-SPME: direct immersion SPME; IT-SPME: in-tube SPME.

虽然GO对含氮、氧官能团的有机物具有良好的吸附性能,但要实现对目标分析物的高选择性萃取,还需要对其进一步修饰。一些芳香族二胺的聚合物,如苯二胺和二氨基吡啶,在聚合物链中含有-NH_2_和-NH-,因为它们可以通过络合作用对酸性化合物或重金属离子(如Pb^2+^和Cd^2+^)提供满意的界面相互作用,从而实现选择性吸附,因此被认为是GO的良好改性剂,同时GO克服了聚合物比表面积小的缺点,可以获得较高的吸附容量。Kojidi等^[18]^制备了GO基聚(2,6-二氨基吡啶)复合材料,并将其装填SPE柱,针对Cd^2+^建立了低检出限(LOD, 0.47 μg/L)和宽线性范围(2~100 μg/L)的分析方法,并用于自来水、海水和废水中Cd^2+^的测定和分析。Farajvand等^[19]^合成了GO/聚苯胺(PANI)纳米复合物作为SPE的吸附剂,并与分散液液微萃取联用,富集Cd^2+^。结合火焰原子吸收光谱分析,建立的方法对Cd^2+^具有低LOD(0.1 μg/L)、LOQ(0.4 μg/L),以及宽线性范围(0.4~1000 μg/L)和高富集倍数(210)。该方法被成功应用到实际样品(矿泉水、河水、自来水)中Cd^2+^的定量检测,回收率为91%~107%。Baranik等^[20]^合成了Al_2_O_3_/GO纳米复合材料,并将其用作D-μ-SPE的吸附剂来萃取水样中的As^5+^和Cr^3+^。在过滤分离后,结合能量色散X射线荧光光谱法,建立了性能良好的分析方法(对As^5+^的LOD为0.02 ng/mg,最大吸附量为43.9 mg/g;对Cr^3+^的LOD为0.11 ng/mg,最大吸附量为53.9 mg/g)。该方法被应用到自来水中As^5+^和Cr^3+^的检测。Amiri等^[21]^以多钨酸阴离子功能化GO为载体制备了纳米杂化材料吸附剂。该吸附剂被应用于D-μ-SPE,基于GO和多氧钨酸阴离子的协同作用机理,高效萃取了非甾体抗炎药。与高效液相色谱-紫外(HPLC-UV)检测联用,建立了一种高灵敏度(LOD, 0.02~0.03 μg/L)、宽线性范围(0.08~200 μg/L)和高富集倍数(2150~2212)的分析方法。该方法被用于自来水、河水和废水中痕量非甾体抗炎药的检测。

Tang等^[10]^制备了GO@NH_2_@Fe_3_O_4_,将其作为MSPE吸附剂,结合基质辅助激光解吸-电离飞行时间质谱(MALDI-TOF-MS),发展了一种分析12种喹诺酮类药物的分析方法。该方法只需不到1 min即可完成样品的分析。Liu等^[22]^以Fe_3_O_4_-GO为载体,制备了磁性铜基MOF(M-MOF-199),并将其用作5种三唑类农药的MSPE吸附剂。由于与三唑类农药中基团之间的相互作用,M-MOF-199表现出良好的萃取性能。基于M-MOF-199-Fe_3_O_4_-GO建立的HPLC-MS/MS分析方法用于测定环境水中三唑类农药,获得了低LOD(0.05~0.1 μg/L)和宽适用范围(0.25~1000 μg/L)。Zang等^[23]^制备了HP-*β*-CD-rGO接枝在磁性Fe_3_O_4_微球上,并将其用于萃取水中Cd^2+^。结合火焰原子吸收光谱,建立了低LOD(0.23 μg/L)和宽线性范围(0.50~100.0 μg/L)的检测方法,并应用于实际样品分析。多巴胺可以通过自聚合在不同基质(贵金属、纳米材料、氧化物和合成聚合物)的表面形成一层薄而均匀的聚多巴胺(PDA)涂层。PDA涂层中含有大量的-OH和-NH_2_,有利于后续的化学改性和材料亲水性的提高。Huang等^[24]^制备了Fe_3_O_4_@G/PDA复合物材料,并用作环境水中4种苯甲酰脲类杀虫剂的吸附剂,通过HPLC-DAD测定杀虫剂的浓度。Chen等^[25]^合成了PDA功能化磁性G和CNTs的杂化纳米复合物作为MSPE吸附剂,并应用于从水样中预富集16种多环芳烃(PAHs)。利用PDA、G和CNTs的良好吸附性能,针对PAHs建立了分析方法,其LOD为0.1~3.0 ng/L,线性范围为10~500 ng/L,线性相关系数为0.9958~0.9989。

分子印迹技术是一种根据模板分子特征建立特定结合位点的技术,它可以很好地匹配模板分子的形状、大小和官能团,进而选择性地识别模板分子。然而,分子印迹技术在实际应用中也存在一些不可避免的缺陷,如结合位点不均匀、传质速度慢、后处理过程复杂等。表面分子印迹技术可以解决上述问题,因为它可以在载体表面形成识别位点,从而获得较高的吸附容量。GO具有优异的力学性能和丰富的含氧官能团等优点,被认为是制备分子印迹聚合物(MIPs)的理想基质材料。以邻苯二甲酸二丙酯和邻苯二甲酸二异辛酯作为双模板剂,利用mSiO_2_@GO@Fe_3_O_4_为载体,制备分子印迹材料作为MSPE吸附剂,同时富集分离6种典型的邻苯二甲酸酯(PAEs),然后用GC-MS进行测定^[26]^。建立的分析方法LOD低(0.01~0.05 μg/L),可适用线性浓度宽(1~50 μg/L),可用于检测和选择性去除水中PAEs。Tian等^[27]^以多巴胺为功能单体和交联剂,微囊藻毒素LR为模板,合成了GO@Fe_3_O_4_-MIPs,并用于水样中微囊藻毒素LR的富集。

也有研究曾通过电化学剥落石墨笔芯制备G涂层,作为SPME纤维^[28]^。由于G与PAHs之间的*π-π*堆积和疏水作用,该涂层在实际样品中表现出良好的萃取效果。G片之间由于*π-π*堆积和范德华力,容易发生不可逆聚集或再堆积。幸运的是,3D-GO中的部分重叠可以防止大横向尺寸柔性G片的聚集,从而形成相互连接的海绵结构。此外,3D-GO除了具有2D-G的固有特性外,还具有孔结构可调、内部空间大、传质速度快等优点,是一种良好的吸附材料。在此基础上,Pei等^[29]^将PANI引入3D-GO的网络中,通过电聚合工艺合成了一种3D-rGO-PANI,然后将其用于固相微萃取,通过负电晕放电电离离子迁移谱法进行检测,测定水样中的乙硫磷。

本课题组^[30]^也开展了一系列GO萃取材料的研究工作。采用1-甲基-3-[3(三甲氧基硅基)丙基]咪唑氯盐作为交联剂,通过层层自组装策略将GO涂层修饰到镀银的不锈钢丝上。通过IL交联剂的化学键合作用以及咪唑阳离子与GO之间的*π-π*和静电相互作用,提高了GO涂层与不锈钢基体之间的结合强度。基于GO涂层的疏水性和芳香结构,该纤维对几种PAHs(芴、蒽、荧蒽、1,2-苯并菲、苯并(*a*)芘)表现出良好的萃取效率,采用直接浸入式萃取和GC检测,经过一系列条件的优化,在最佳条件下建立了分析方法,具有宽的线性范围(0.5~200 μg/L)和低的LOD(0.05~0.10 μg/L),最终将该方法应用于雨水和河水等环境水样的分析检测。为了提高G涂敷纤维的使用寿命和化学稳定性,我们通过化学镀和化学键合技术研制了一种新型GO涂层键合SPME纤维^[31]^,再还原为G涂层,并将其应用到5种正构烷烃(正十一烷、正十二烷、正十三烷、正十四烷和正十六烷)的前处理中。通过GO掺杂1-(3-氨基丙基)-3-(4-乙烯基苄基)咪唑-4-苯乙烯磺酸盐单体与1,6-二-(3-乙烯基咪唑)己烷-六氟磷酸盐交联剂的共聚反应,我们制备了GO增强的聚合ILs整体柱^[32]^。结合HPLC法,该整体柱作为SPE吸附剂对水样中的几种酚类化合物实现了富集。我们还采用电沉积法将GO修饰到碳纤维(CFs)表面,并将GO-CFs作为萃取材料填充到聚醚醚酮(PEEK)管中用于管内固相微萃取,并与HPLC连接,建立在线SPME-HPLC分析系统。以10种PAHs为分析物,对重要萃取条件进行了优化,建立了在线分析方法,方法线性范围宽(0.01~50 μg/L),灵敏度高(LOD, 0.001~0.004 μg/L),富集效应强(1133~3840),分析速度快且重复性好,被成功应用于废水样品中PAHs的在线测定^[33]^。

综上所述,G和GO基复合材料在样品前处理中已被广泛应用于环境样品中多类别分析物如重金属离子、抗生素、农药、多环芳烃、塑化剂等的高效富集,并表现出优异的萃取性能。

### 1.2 碳纳米管

CNTs被分为SWCNTs和MWCNTs两种,由于其独特的优势结构和物理化学性质,如高孔隙率、中空结构、能够与分子形成*π-π*和范德华力等多重相互作用,并易于功能化,引起了人们的广泛关注^[34]^。基于此,CNTs作为一种新型的吸附剂在分析化学中得到了广泛应用,并展示出对非极性到强极性多种有机和无机分析物的良好萃取潜力^[35]^。有机物在CNTs上的吸附机理主要受疏水作用和*π-π*相互作用控制,因而CNTs与分析物的萃取选择性差,对某些分析物萃取能力低。值得一提的是,CNTs外表面丰富的离域*π*电子可以作为通过*π-π*相互作用捕获芳香族分析物的基础。此外,CNTs中的碳原子是*sp*^2^和*sp*^3^的杂化形式,利于产生-COOH、-OH或-C=O等基团进行功能化。因此,CNTs的改性能进一步为不同的分析物提供选择性吸附的相互作用^[36]^。研究人员已利用有机官能团、纳米材料、MOFs、聚合物和MIPs对CNTs进行了修饰,赋予其各种萃取机理,包括氢键、静电力、配位、分子尺寸选择效应等。表2汇总了碳纳米管样品前处理材料在环境污染物分析检测中的部分最新应用进展。

**表2:** 碳纳米管基样品前处理材料在环境污染物分析检测中的应用

Adsorbent	Analytes	Sample	LOD	Linear range	Analytical method
MWCNTs^[34]^	atrazine	water	0.66 μg/L	2-100 μg/L	SPE-BCAE-HPLC-UV
HCl-treated MWCNTs^[37]^	thirteen pharmaceuticals and two metabolites of metamizole	water	0.2-103 ng/L	10-250 μg/L	SPE-HPLC-MS/MS
ox-MWCNTs^[38]^	three progestins	water	0.05-0.14 μg/L	0.90-9.0 μg/L	SPE-HPLC-UV
3D MWCNTs@g-C_3_N_4_@ Fe_3_	sixteen PAHs	water	0.001-0.5 μg/L	0.2-200 μg/L	MSPE-GC-FID
M-M-ZIF-67^[40]^	nine organochlorine pesticides	agricultural water	0.07-1.03 μg/L	1-200 μg/L	MSPE-GC-MS/MS
MMP/ZIF-8^[41]^	five triazole fungicides	water	0.08-0.27 μg/L	1-400 μg/L	MSPE-GC-MS/MS
MFCA^[42]^	nine perfluorocarboxylic acids and perfluorosulfonic acids	water	0.010-0.50 ng/L	0.4-10000 ng/L	MSPE-HPLC-MS/MS
oxidized MWCNTs^[43]^	four PAHs	water	2-20 ng/L	10-500 ng/L	HS-SPME-GC-MS
oxidized-CNTs^[44]^	menthol	water	20 μg/L	50-100000 μg/L	HS-SPME-GC-FID
MWCNTs/NaDC^[45]^	five phenols	seawater	0.15-0.30 μg/L	1-100 μg/L	SPME-HPLC-UV
CNT/magnetite/PA^[46]^	four phenols	water	0.008-0.07 μg/L	0.01-500 μg/L	SPME-GC-MS
MNC^[47]^	glucocorticoid	water	0.0075-0.16 ng/L	0.05-1000 ng/L	MSPE-HPLC-MS/MS
CNT-Ti	seven PAHs	water	0.002-0.004 μg/L	0.01-200 μg/L	SPME-GC

MWCNTs: multi-walled carbon nanotubes; ox-MWCNTs: oxidized MWCNTs; 3D MWCNTs@g-C_3_N_4_@Fe_3_O_4_: three-dimensional MWCNTs@graphitic carbon nitride@Fe_3_O_4_ nanocomposites; M-M-ZIF-67: zeolitic imidazolate framework based on magnetic multiwalled carbon nanotubes; MMP/ZIF-8: magnetic zinc-based zeolitic imidazolate framework; MFCA: magnetic fluorinated carbon nanotubes adsorbent; MWCNTs/NaDC: MWCNTs/sodium deoxycholate; CNT/magnetite/PA: nanotube/magnetite/polyaniline; MNC: magnetic nanocomposite; CNT-TiO_2_: composite between carbon nanotubes and titanium oxide; BCAE: bio-coacervation extraction.

Abbasi等^[34]^发展了一种基于MWCNTs的固相萃取与生物凝聚萃取相结合的样品前处理方法。该方法主要分为两个萃取步骤:第一步,MWCNTs萃取分析物,然后进行洗脱;第二步,使用生物表面活性剂和白介素组成的生物聚集体对分析物进行再富集,并借助HPLC-UV对环境水中痕量阿特拉津进行分析,回收率为90.1%~97.2%,结果令人满意。对MWCNTs进行化学处理可以改变其表面孔隙率和含氧官能团的数量,这些参数会影响MWCNTs的吸附性能。Lalovic等^[37]^分别利用硝酸、氢氧化钾和盐酸处理MWCNTs,结合HPLC-MS/MS,比较了处理前后的材料对水样中13种药物及2种代谢物的萃取能力。结果表明,HCl处理后的MWCNTs减少了电子受体基团的数目,有利于提高它的萃取效率,从而对药物吸附产生积极影响。分析方法具有低LOD(0.2~103 ng/L)和良好的线性范围(10~250 ng/mL),适用于分析包括地表水和地下水等环境水样。使用氧化的MWCNTs作为吸附剂,提出了一种流动注射辅助的在线SPE方法,从环境水中同时萃取和测定左炔诺孕酮、甲羟孕酮和炔诺酮^[38]^。与传统吸附剂相比,ox-MWCNTs表现出更高的吸附容量和更好的吸附-解吸动力学性能,并且-OH和-COOH的引入增强了MWCNTs在水溶液中的分散性和选择性。

为了解决Fe_3_O_4_纳米粒子占据纳米管表面而导致MWCNTs吸附容量降低的问题,Zhang等^[39]^引入了具有类似G结构的g-C_3_N_4_来制备3D纳米复合物作为MSPE吸附剂。利用*π-π*、氢键和静电作用在内的多重相互作用,3D MWCNTs@g-C_3_N_4_@Fe_3_O_4_吸附剂对PAHs具有良好的吸附性能。此外,建立的MSPE-GC-FID方法的线性范围为0.05~100 μg/L, LOD为0.001~0.5 μg/L,且重复性好(RSD≤5.0%),可用于自然水体中16种PAHs的检测。

沸石咪唑酯骨架结构材料(ZIFs)作为MOFs的一种,具有水稳定性好、微孔率高、空腔结构均匀等优点。Huang等^[40]^引入ZIFs,通过有机-无机配位制备了多孔磁性MWCNTs复合物(M-M-ZIF-67)吸附剂,并将其用于MSPE。结合GC-MS/MS检测系统,建立了测定农业灌溉水中有机氯农药的分析方法,M-M-ZIF-67对有机氯农药的吸附能力是Fe_3_O_4_-MWCNTs的近3倍。采用相同的合成方法,基于Fe_3_O_4_/MWCNT@PDA,他们还合成了磁性MWCNTs/ZIF-8吸附剂^[41]^,建立了MSPE-GC-MS/MS方法来检测环境水中的三唑类杀菌剂。该方法具有LOD低(0.08~0.27 μg/L)、线性范围宽(1~400 μg/L)以及线性好(相关系数≥0.9915)等优势。

磁性MWCNTs通过各种亲水性基团(如-COOH和-OH)的功能化,提高它在水样中的分散性。Huang等^[42]^采用一锅水热法制备了富氟磁性氟化CNTs吸附剂(MFCA)。通过氟-氟、疏水、氢键等多种相互作用,MFCA-MSPE对具有高氟性质的全氟烷基羧酸和全氟烷基磺酸表现出良好的萃取性能。与HPLC-MS/MS相结合,基于MFCA/MSPE的方法获得了低LOD(全氟烷基羧酸为0.010~0.036 ng/L,全氟烷基磺酸为0.024~0.50 ng/L)、宽线性适用范围(0.4~10000 ng/L)和满意的精密度(RSD≤10%),适用于自来水、河流、湖泊和废水中痕量目标分析物的定量检测。

溶胶-凝胶技术实现了一步制备具有高热/化学稳定性的化学键合涂层。在此基础上,Mohammadiazar等^[43]^制备了聚二甲基硅氧烷(PDMS)/二乙烯基苯/MWCNTs纤维,并首次用ox-MWCNTs对其进行改性,以改善纤维基体的粗糙度,便于溶胶-凝胶工艺的进行。以PAHs为分析物,优化了一系列主要影响因素,与GC-MS联用建立了检测水样中PAHs的方法。Yarazavi等^[44]^使用溶胶-凝胶制备方法,发展了一种用于SPME的TiO_2_溶胶-凝胶涂层,使用钛酸异丙酯和3-(三乙氧基硅丙基)胺作为前体,并在制备过程中加入ox-CNTs。将该纤维与GC-FID联用,建立了水溶液中顶空萃取薄荷醇的方法,该方法的灵敏度(0.02 μg/mL)和检测适用范围(0.05~100 μg/mL)令人满意。

为了提高CNTs的分散性,改变CNTs的结构和极性,表面活性剂或大分子对CNTs进行修饰也是一种有效的方法。利用这一策略,Zhou等^[45]^制备了脱氧胆酸钠功能化MWCNTs涂层纤维,并将其结合HPLC测定,用于南海海水和废水中痕量酚的富集分析。由于MWCNTs/脱氧胆酸钠表面亲水基团(-OH和-COOH)与酚类物质之间的氢键作用,使得该纤维对酚类物质的吸附能力优于商用PDMS/二乙烯基苯纤维。

Tafazoli等^[46]^通过共沉淀法在CNTs表面制备单分散的磁铁矿纳米球,并对该复合材料表面进行PANI纳米层原位改性,合成了高效的有机-无机CNTs/磁铁矿/PANI纳米复合材料。将其修饰到不锈钢丝表面制备SPME纤维,用于萃取酚类化合物,与GC-MS相结合,建立了低检出限(0.008~0.07 μg/L)和宽线性范围(0.01~500 μg/L)的分析方法,并应用于实际水样的分析。Huang等^[47]^以氨基衍生化CNTs(CNTs-NH_2_)和1-甲基咪唑修饰GO为原料,采用一步水热法合成了一种新型富官能团的磁性纳米复合材料(MNC)。它结合了CNTs和GO的优点,同时具有丰富的氨基和咪唑基团,利用MSPE可以高效富集河水、湖水、海水和污水中的超微量糖皮质激素,检出限可以低至0.0075~0.16 ng/L,线性范围宽至0.05~1000 ng/L。

本课题组^[48]^采用化学镀和溶胶-凝胶技术,制备了一种新型CNTs-TiO_2_复合涂层键合不锈钢丝SPME纤维,借助于GC-FID检测,研究了7种PAHs在纤维上的吸附行为。在优化的萃取条件下,对SPME-GC分析方法进行评价。该方法获得了高的灵敏度(LOD, 0.002~0.004 μg/L)和好的线性范围(0.01~100和0.01~200 μg/L)。该纤维具有很高的热稳定性(300 ℃)和优异的耐酸碱稳定性。将建立的分析方法用于实际水样的分析,得到了满意的结果。

综上,碳纳米材料作为一种性能优良的吸附剂,在样品前处理中有着广泛的应用前景,它既具有碳材料的稳定性,又具有纳米材料的优异吸附性,有助于成为对其他材料进行改性的良好平台。碳纳米材料与其他材料结合可以产生多种吸附机制,包括氢键、*π-π*堆积、静电、疏水和亲水作用,拓展了各自的应用范围。尽管碳纳米材料与MOFs、MIPs、磁性材料等纳米材料的结合已有报道,但仍有较大的发展空间。碳纳米材料在不同复杂基体中的广泛应用仍然是一个挑战。总之,碳纳米材料在前处理领域有着巨大的发展潜力,需要进一步推进新材料的设计和制备,探索碳纳米材料更广泛的应用。

## 2 气凝胶

气凝胶是一种纳米多孔轻质固体材料,具有独特的连续三维(3D)网络结构,同时具有低密度、高表面积、低导热率和丰富孔隙^[49,50]^,根据其化学组成可以分为无机气凝胶、有机气凝胶及复合气凝胶。气凝胶自1931年出现以来,因其出色的吸附性质而吸引了众多关注,并被引进样品前处理领域^[51]^。

### 2.1 无机气凝胶

炭气凝胶是无机气凝胶中的一类,通常利用有机气凝胶高温碳化获得^[52]^,其将较高表面积和纳米级孔径结构相结合,使得炭气凝胶展示出强大的吸附性能。Joul等^[53]^热解5-甲基间苯二酚和甲醛合成的有机气凝胶,直接获得炭气凝胶。该气凝胶具有丰富的孔隙,被装填进注射器制备成SPE柱,用于硫芥子降解产物的萃取,建立了SPE-HPLC-DAD分析方法。该方法的LOD为0.17~0.50 μmol/L,分析物的加标回收率为79.8%~115.1%。结果表明炭气凝胶SPE柱可以快速、灵敏地富集水样中痕量化学战剂降解产物。此外,高温碳化生物质材料是获取炭气凝胶的另一种方式。本课题组^[54]^将柚子皮内部的海绵组织通过冷冻干燥保持其多孔形态,然后在氮气保护下经高温碳化,制成新型生物质炭气凝胶。该材料成本低,可再生,制备过程不涉及有机反应,避免了环境污染及对人体的伤害。将其应用于实际水样中PAHs的检测,建立了IT-SPME-HPLC-DAD在线分析方法,萃取性能可以与其他萃取材料相媲美,富集倍数高达3425, LOD低至0.005 μg/L。

有机磷农药作为一种广泛应用于农业生产的杀虫剂,主要用于防治植物病、虫、草害等,滥用或误用均会对环境产生危害,并威胁人体健康。为了对有机磷农药进行有效检测,最近,Jafari等^[55]^以苹果作为原料,制备出了纳米结构的海绵状生物炭气凝胶,利用溶剂辅助器液相微萃取结合离子迁移谱实现了对其的有效检测,检出限低至0.09 μg/L。Joul等^[56]^则将炭气凝胶直接原位制备在不锈钢丝上,制成萃取纤维对有机磷农药进行萃取。由于所制备炭气凝胶高的比表面积(501.157 m^2^/g)以及与目标分析物之间的*π-π*共轭和其他一些相互作用,这种萃取纤维表现出了比商业涂层纤维更强的萃取能力。Dong等^[57]^则以ILs作为模板剂,引入到间苯二酚-甲醛(RF)气凝胶的合成反应中,制备得到IL-RF气凝胶,对其进行高温碳化处理,制备出了一种新型的炭气凝胶。通过这种方式不仅避免了冷冻和超临近干燥,而且所获得的炭气凝胶同时具有介孔和交联结构。对其进行羧基化处理后,作为涂层应用于SPME纤维的制备,结合HPLC-UV分析,实现了对养殖场污水样品中6种四环素的有效检测。

石墨烯气凝胶(GA)是一种由石墨烯构成的气凝胶材料,具有3D结构及快速传质的特征,是一种优异的吸附材料。Han等^[58]^采用无模板溶胶-凝胶-冷冻干燥法制备了GA材料,将其填进注射器管内制成SPE柱,通过推拉手柄可以实现快速富集目标分析物,从实际水样中富集内分泌干扰物(EDC)和多氯联苯(PCB),分别通过HPLC和GC-MS进行检测。获得了较低LOD(EDC为0.01~0.11 ng/mL; PCB为0.19~1.53 ng/L)及满意的加标回收率(76.3%~112.5%)。此外,他们还将该萃取柱用于富集拟除虫菊酯,获得了令人满意的结果,这进一步扩大了它的应用范围^[59]^。Sun等^[60]^利用GA-SPE柱萃取水样中的6种有机磷农药,结合GC-MS进行分析,建立了线性范围宽(0.5~500 μg/L)、LOD低(0.12~0.58 μg/L)的方法,用于测定河流、湖泊、饮用水中的农药,相对加标回收率为93.8%~104.2%。GA不仅可以用作SPE柱的吸附剂,还可以用作分散SPE的吸附剂^[61]^。由于*π*共轭结构和丰富的孔径,Wang等^[61]^建立了土壤中6种氯酚的SPE-HPLC-UV分析方法,LOD为0.02~0.10 μg/L,在相同的萃取条件下,GA的萃取能力要比商品化C18和SWCNTs高。由于GA的疏水性,上述所有方法均用于萃取疏水性污染物,但不适用于高效萃取水溶性分析物。为了改善这个问题,Tang等^[62]^利用聚乙烯醇作为交联剂,合成一种可压缩且具有两亲性的G气凝胶,装填进注射器针管内用于河水中苯酚的萃取。河水的加标回收率为96.3%~102.4%, RSD为1.8%~5.4%,表明该材料对实际样品中的苯酚具有特异性吸附。而且聚乙烯醇的亲水性使得气凝胶可以高效富集目标分析物,而且提高了可重复使用次数。

此外,模板法也被应用于GA的制备。Ding等^[63]^使用聚苯乙烯微球作为模板剂制备了一种分级多孔GA吸附剂,用于水样中7种拟除虫菊酯的萃取。首先在高速搅拌下混合GO和聚苯乙烯微球溶液,并使用微型注射器吸取混合溶液进行冷冻干燥处理,获得GO/聚苯乙烯气凝胶;之后高温肼蒸汽不断通过注射器完成还原反应,原位生成G/聚苯乙烯气凝胶;再使用甲苯洗去模板剂得到分级多孔的GA。以该气凝胶为吸附材料,对河水中的拟除虫菊酯进行富集萃取,采用GC-MS分析。由于高的比表面积以及强的疏水性,该吸附剂具有出色的萃取性能,可在一个推拉过程内完成分析物吸附。此外,对于大多数分析物,出现了显著的化学稳定性(pH范围:3~9),良好的线性关系(0.2~50 μg/L)和较低的LOD(0.012~0.11 μg/L)。

复合型气凝胶往往具有单个材料所不具备的优势。例如壳聚糖作为一种廉价的天然生物材料,可以通过强静电作用与氢键作用增强GA的机械性能。Gao等^[64]^就利用这种特性制备了G/壳聚糖复合气凝胶,并将其作为萃取材料结合SPE-GC-MS方法用于PAHs的检测。该复合材料具有高的比表面积和蜂窝状结构,能够提供*π-π*共轭、氢键和范德华力等作用力,而且弹性和多孔的结构也显著减少了所需洗脱液的体积和压力。实验也证明该方法能够提供低检出限(1.7~8.8 pg/mL)与宽线性范围(10~2000 pg/mL),该材料是一种有潜力的萃取材料。此外,Sun等^[65]^采用羧基化的MWCNTs与氧化石墨烯混合进行化学还原,并冷冻干燥制备具有3D结构的CNTs/GO复合气凝胶,也实现了对环境水样中有机磷农药的高效萃取。

炭气凝胶的吸附能力优异,但是不易回收,这限制了其发展。通过将磁性纳米材料结合到GA中,获得了一种磁性G气凝胶(Fe_3_O_4_/GA)。磁性纳米颗粒嵌入气凝胶骨架中,赋予材料强磁性特征的同时也增加了吸附位点,利于其高容量萃取目标分析物,已成功用于水样中双酚A的萃取^[66]^。

SiO_2_气凝胶作为一种代表性的无机气凝胶,除了具有高比表面积外还拥有良好吸附性质、3D空间网络结构、可调控和易于化学改性等特点。本课题组^[67]^将SiO_2_气凝胶粉末涂覆在不锈钢丝表面上。然后将其置于PEEK管中,得到SPME管,并联用HPLC-DAD进行进一步分析。比较极性不同的3种类别的分析物(PAHs、雌激素和PAEs)的萃取效果,由于极性作用和氢键相互作用,该气凝胶涂层显示出对PAHs的高吸附能力。经过优化萃取和脱附条件后获得分析方法,成功用于检测实际水样中低浓度的PAHs,富集倍数可达576~2245, LOD为0.005~0.050 μg/L。但是SiO_2_气凝胶也存在机械强度低、稳定性差、选择性不理想的问题。Roostaie等^[68,69]^使用三乙基氯硅烷和三甲基氯硅烷对SiO_2_气凝胶进行功能化,这些硅烷中的疏水基团代替了SiO_2_气凝胶表面的-OH,从而获得了疏水性SiO_2_气凝胶,通过顶空针阱萃取形式富集水中的氯苯。通过优化萃取条件,这些分析方法均展示出良好的线性和较低的LOD,并且可以满足分析水样中此类污染物的要求。

基于SiO_2_气凝胶存在的问题,本课题组^[70]^做了一系列研究工作。打破原来单一前驱体制备SiO_2_气凝胶的常规方法,利用3-氨丙基三甲氧基硅烷与3-氯丙基三甲氧基硅烷反应合成了疏水性的硅烷偶联剂三-[3-(三甲氧基硅基)丙基]胺,它和正硅酸四乙酯作为共前体,制备获得了有机杂化SiO_2_气凝胶。将该气凝胶涂覆到金属丝表面上制成SPME纤维,与GC-FID联用,针对环境中的PAHs建立了一种线性范围为0.005~20 μg/L、LOD为0.001~0.030 μg/L的分析方法。制作SPME涂层的物理涂覆法虽然操作简便,但是涂层不稳定。因此,我们发展了一种化学键合气凝胶涂层,利用对苯二醛对SiO_2_气凝胶进行改性^[71]^,而且气凝胶与玄武岩纤维基体生成-Si-O-Si-键使得涂层可以化学键合在表面,提高了稳定性。制成SPME管用于污水中雌激素的萃取,富集倍数高达3132, LOD为0.01 μg/L。将基于杂化气凝胶的萃取材料依次经过甲醇、酸、碱和盐溶液连续冲洗后,仍保留了良好的萃取效率,表明该研究发展的杂化气凝胶键合涂层材料具有优异的化学稳定性。表3总结了炭气凝胶等无机气凝胶作为样品前处理材料在环境污染物分析检测方面的应用。

**表 3:** 无机气凝胶样品前处理材料在环境污染物分析检测中的应用

Adsorbents	Analytes	Samples	LODs	Linear ranges	Analytical methods
CA^[53]^	ten HD	environmental pore water	0.17-0.50 μmol/L	1.0-20 μmol/L	SPE-HPLC-DAD
Biocharcoal aerogel^[54]^	eight PAHs	water, honey and pear syrup	0.005-0.050 μg/L	0.017-15 μg/L	IT-SPME-HPLC-DAD
CA^[55]^	six organophosphorus pesticides	environmental water	0.09 μg/L	-	liquid-phase microextraction-SESI-IMS
CA^[56]^	six organophosphorus pesticides	environmental water	0.11-0.83 μg/L	-	SPME-GC-MS
IL-CA^[57]^	tetracyclines	water	0.36-0.71 μg/L	2-1000 μg/L	SPME-HPLC-UV
GA^[58]^	three endocrine disrupting chemicals and seven polychlorinated biphenyls	river, lake, drinking and tap water	0.01-0.11 μg/L, 0.19-1.53 ng/L	0.05-100 μg/L, 0.01-5 μg/L	SPE-HPLC and SPE-GC-MS
GA^[59]^	five pyrethroids	drinking water	0.83-9.31 ng/L	0.02-10 μg/L	SPE-GC-MS
GA^[60]^	six organophosphorus pesticides	river water	0.12-0.58 μg/L	0.5-500 μg/L	SPE-GC-MS
GA^[61]^	six chlorophenols	soil	0.02-0.10 μg/L	50-1000 μg/L	MSPD-HPLC-UV
GA^[62]^	eight phenols	river water	0.016-0.075 μg/L	0.05-40 μg/L	in-syringe SPE-HPLC-UV
GA^[63]^	six pyrethroids	river water	0.012-0.11 μg/L	0.2-50 μg/L	in-syringe SPE-GC-MS
GCA^[64]^	eight PAHs	river, tap water	1.7-8.8 ng/L	10-2000 ng/L	SPE-GC-MS
C-MWCNT-GA^[65]^	six organophosphorus pesticides	wetland, lake, and river water	0.28-0.52 μg/L	0.96-1.64 μg/L	SPE-GC-MS
Silica aerogel^[67]^	eight PAHs	bottled water, tap water, river water and tea water	0.005-0.050 μg/L	0.017-15 μg/L	IT-SPME-HPLC-DAD
Trimethylchlorosilane modified nanoporous silica aerogel^[68]^	four chlorobenzenes	water	0.4-0.8 ng/L	3-3000 ng/L	headspace needle trap extraction-GC-MS
Triethylchlorosilane modified nanoporous silica aerogel^[69]^	four chlorobenzenes	water	0.3-1 ng/L	3-3000 ng/L	headspace needle trap extraction-GC-MS
Organic-inorganic hybrid silica aerogel^[70]^	eight PAHs	water	0.001-0.030 μg/L	0.005-20 μg/L	fiber SPME-GC-FID
Organically modified silica aerogel^[71]^	five estrogens	sewage and emollient water	0.01-0.05 μg/L	0.03-100 μg/L	IT-SPME-HPLC-DAD

CA: carbon aerogel; IL-CA: ionic liquid-carbon aerogel; GA: graphene aerogel; GCA: graphene/chitosan composite aerogel; C-MWCNT-GA: three-dimensional carboxylated multi-walled carbon nanotubes-graphene aerogel; HD: sulfur mustard; SESI-IMS: secondary electrospray ionization-ion mobility spectrometry.

### 2.2 有机气凝胶

Pekala等^[72]^成功合成的RF气凝胶标志着有机气凝胶的开始,之后相继出现许多其他种类的有机气凝胶,如三聚氰胺-甲醛(MF)气凝胶、酚醛树脂-糠醛气凝胶。不同于小分子聚合的无机气凝胶,有机气凝胶通常是有机物单体或低聚体溶于溶剂中,经过化学反应,生成链状或无序枝状网络结构,最后经溶胶-凝胶过程实现凝胶化,溶剂置换后,再经干燥除去溶剂得到干燥后的气凝胶。在兼具比表面积大、孔隙率高、吸附能力强等特点之外,有机气凝胶表现出更高的机械性能,推动了气凝胶的发展^[72]^。

MF气凝胶作为一种代表性的有机气凝胶,能够与化合物产生*π-π*和范德华力等相互作用,已被应用于样品前处理领域,尤其对疏水性有机环境污染物展示出良好的富集能力。本课题组^[73]^在MF气凝胶制备过程中加入玄武岩纤维,使得气凝胶原位生长在纤维表面,将其填充进PEEK管,研究其对PAHs的萃取效果^[73]^。基于MF气凝胶的IT-SPME-HPLC在线分析方法呈现出宽线性范围(0.06~30 μg/L)和低检出限(0.01~0.05 μg/L)。考虑到纳米材料引入气凝胶能够实现对其性能的改进,我们选择具有大比表面积和稳定化学性质的氮化硼纳米片(BNNs)来杂化MF气凝胶^[74]^。借助于BNNs的共轭体系及其疏水性,使得BNNs-MF气凝胶对PAHs表现出良好的萃取性能,LOD低至0.005 μg/L,萃取方法的引入将色谱仪器的检测灵敏度提高了3个数量级。此外,除了对气凝胶进行简单的物理掺杂改性之外,表面聚合和化学键合方法也已用于功能化MF气凝胶^[75,76]^。多巴胺的自聚合作用使其在任何基材上易于形成均匀的涂层^[77]^,基于此,我们制备了PDA功能化的MF气凝胶,并将其用于检测地表水中的PAEs^[75]^。PDA不仅可以使MF气凝胶牢固地黏附在玄武岩纤维的表面,而且可以提供更多的吸附位点。将纤维填充的萃取管与HPLC在线联用,富集倍数高达2221, LOD为0.02~0.05 μg/L。为了提高MF气凝胶的萃取选择性,丰富其实际应用范围,还使用1-十二烷基-3-(3-氨基丙基)咪唑溴盐ILs改性MF气凝胶,富集地表水样品中5种雌激素污染物。结果表明,ILs改性MF气凝胶在分析物的萃取中具有良好的特异性,并且在样品中得到了满意的加标回收率(80%~120%)^[76]^。此外我们还利用RF气凝胶作为吸附剂,将该气凝胶原位制备到玄武岩纤维上作为萃取涂层,用于萃取和分析雌激素类污染物。氢键和疏水作用使气凝胶表现出出色的萃取性能,获得了良好的线性范围(0.017~20 μg/L),较低的LOD(0.005~0.030 μg/L)和令人满意的重复性(RSD<2.7%)^[78]^。表4总结了有机气凝胶作为样品前处理材料应用于环境污染物的分析检测。

**表4:** 有机气凝胶样品前处理材料在环境污染物分析检测中的应用

Adsorbent	Analytes	Samples	LOD/(μg/L)	Linear range/(μg/L)	Analytical method
MF aerogel^[73]^	eight PAHs	rain and tap water	0.01-0.05	0.06-30	IT-SPME-HPLC-DAD
BNNs/MF aerogel^[74]^	eight PAHs	rain and soil solution	0.005-0.010	0.016-20	IT-SPME-HPLC-DAD
PDA-MF aerogel^[75]^	seven PAEs	surface water	0.02-0.05	0.07-30	IT-SPME-HPLC-DAD
IL modified MF aerogel^[76]^	five estrogens	water, aloe	0.05-0.20	0.15-20	IT-SPME-HPLC-DAD
RF aerogel^[78]^	five estrogens	water	0.005-0.030	0.017-20	IT-SPME-HPLC-DAD
Al(Ⅲ)-MOA^[79]^	BTEX, five phenols	water	-	-	HS-SPME-GC-MS
MOA^[80]^	five chlorobenzenes	river and tap water,	0.0001-0.06	0.0004-20	HS-SPME-GC-ECD
		sludge and coastal soil			

MF aerogel: melamine-formaldehyde aerogel; BNNs: boron nitride nanosheets; PDA: polydopamine; IL: ionic liquid; RF aerogel: resorcinol-formaldehyde aerogel; Al(Ⅲ)-MOA: Al(Ⅲ)-carboxylate metal-organic aerogels; BTEX: benzene, toluene, ethylbenzene and xylene; ECD: electron capture detection.

早在2013年,研究人员就使用金属配位来促进凝胶过程。Li等^[79]^利用轻金属Al(Ⅲ)和羧酸逐渐组装而成的金属有机凝胶(MOG)合成了多层多孔金属有机气凝胶(MOA)。选择微孔晶体MOFs颗粒作为MOG的构建单元,在此过程中,MOFs可以并入中/大孔气凝胶支架中,从而在气凝胶中形成有序的颗粒内微孔和颗粒间中孔。结合了MOFs和轻质气凝胶优势的Al(Ⅲ)-MOA被用作SPME纤维涂层,用于在顶空萃取模式下检测苯系物和酚类化合物。与100 μm的PDMS和聚酰胺纤维相比,Al(Ⅲ)-MOA纤维在多次循环后显示出更高萃取效率、更短萃取时间和更好的重复使用性。这项工作提出了一种合成层状多孔有机气凝胶的新方法,并且显示了通过该方法制备的萃取涂层具有高效率、高灵敏度和良好选择性。 此外,Saraji等^[80]^还合成了另一种铁基MOA。MOA涂在镍铬合金丝上,并通过顶空萃取和气相色谱-电子俘获检测器(GC-ECD)分析环境样品(水和土壤污泥)中的氯苯。在最优化的条件下,获得了较宽的线性范围(0.4~20000 ng/L),灵敏的检出限(0.1~60 ng/L)。在MOA涂层和目标分析物的芳环之间存在强的*π-π*相互作用,使得MOA涂层的纤维比商业PDMS纤维具有更高的萃取效率。此外,在50次使用后,纤维仍保持高萃取效率,并且在300 ℃以下具有良好的热稳定性。

## 3 三嗪基材料

三嗪基材料由于优异的物化性质,被广泛应用于色谱分析、化学传感、能源储存、药物缓释等领域。此类材料通常具有高的表面积,而且因其能对化合物提供多种作用,因此在吸附和萃取领域也受到研究者的关注,成为近年来样品前处理方向的热点材料。三嗪基吸附材料分为三嗪基功能化载体材料和三嗪基聚合物材料。聚合物中的共价三嗪框架材料(CTFs)作为共价有机骨架材料(COFs)的一种,具有良好的结晶度、优异的化学稳定性和耐热性,被认为是萃取领域的一种潜在吸附剂材料。本部分主要综述了三嗪基材料在样品前处理领域中应用于环境水样分析的工作(见表5)。

**表 5:** 三嗪基吸附剂作为样品前处理材料在环境污染物分析检测中的应用

Adsorbents	Analytes	Samples	LOD/(μg/L)	Linear range/ (μg/L)	Analytical method
Tetraazacalix[2]arene[2]triazine	five PAHs	river water	0.0004	0.0005-0.1	SPE-HPLC-FLD
bonded silica^[81]^	Cu(Ⅱ)		0.015	0.1-100	SPE-graphite furnace atomic absorption spectrometry
Tetraazacalix[2]arene[2]triazine coated Fe_3_O_4_/Si	five PAHs	surface water and ground water	0.00009-0.00015	0.0005-0.05	MSPE-HPLC-FLD
	six nitroaromatics		0.006-0.011	0.02-0.2	MSPE-HPLC-UV
	four metal ions		0.017-0.053	0.02-2.0	MSPE-atomic absorption spectrometry
Melamine sponge functionalized with urea-formaldehyde co-oligomers^[83]^	ten hydrophobic analytes	lake water	0.01	1.0-100	SPE-HPLC-DAD
Melamine sponge decorated with copper sheets^[84]^	ten sulfonamides	lake water	0.008	0.5-150	SPE-HPLC-DAD
Triazine-based polymeric modified Fe_3_O_4_/GO^[85]^	acidic and basic pesticides	water samples	0.17	5.0-500	MSPE-HPLC-UV
Magnetic covalent triazine-based frameworks^[86]^	six perfluorinated acids	water samples	0.00062	0.005-4.0	MSPE-HPLC-MS/MS
Triazine-cored covalent organic framework^[87]^	five polybrominated diphenyl ethers	water samples	0.00003	0.0001-5.0	DSPE-GC-MS/MS
Covalent triazine-based framework- grafted functionalized fibrous silica sphere^[88]^	chlorpyrifos fenthion	water samples	0.05 0.55	0.1-1 1.0-700	SPME-ion mobility spectrometry
Triazine-based covalent organic framework^[89]^	nine antibiotics	water samples	0.031	1-500	SPE-UPLC-MS/MS
Triazine-based organic polymers@ SiO_2_ nanospheres^[90]^	eight PAHs	water samples	0.003	0.01-20	IT-SPME-HPLC-DAD
Triazine-based covalent porous organic polymer^[91]^	eight PAHs	water samples	0.004	0.013-20	IT-SPME-HPLC-DAD

DSPE: dispersive solid phase extraction.

SiO_2_纳米球作为吸附剂广泛应用于水样中的污染物萃取,然而对有机污染物和重金属同时进行富集的研究十分有限,基于四氮杂杯[2]芳烃[2]三嗪对PAHs产生的*π-π*作用以及对Cu^2+^的螯合作用,将其键合于SiO_2_球表面作为SPE吸附剂^[81]^。对影响萃取效率的条件进行了优化,包括有机溶剂的种类和浓度、样品溶液的pH值、萃取流速和体积。将SPE分别与HPLC-FLD、石墨炉原子吸收光谱进行联用对河水中的PAHs和Cu^2+^进行检测,分析方法具有低LOD(PAHs为0.4 ng/L, Cu(Ⅱ)为15.0 ng/L)、宽线性范围(PAHs为0.25~100 ng/L, Cu(Ⅱ)为0.10~100 μg/L)和良好重现性(RSD<6.4%)。与传统的SPE相比,磁性SPE形式中纳米吸附剂材料能充分与样品溶液中的分析物接触,从而提高萃取效率和富集能力。以四氮杂杯[2]芳烃[2]三嗪包覆的Fe_3_O_4_/SiO_2_磁性纳米粒子实现了对5种PAHs、6种硝基芳烃和4种金属离子的萃取富集^[82]^。三聚氰胺海绵是一种廉价易得的多孔三嗪基聚合物,为提高其表面的疏水性,进行了脲-醛共聚改性^[83]^。该材料被作为吸附剂萃取了10种分析物,优化萃取和脱附条件,借助于HPLC分离和检测,建立了分析方法。该方法LOQ低(0.03~1.0 g/L)、线性范围宽、回收率高(92%~100%)。基于三聚氰胺海绵在上述萃取中取得的良好效果,考虑用化学镀铜对其进行功能化处理^[84]^。所制备的功能化材料用于建立高效灵敏的萃取方法,去吸附湖水和牛奶中的磺胺类药物。该材料表面呈现出疏水性和对磺胺类良好的亲和作用,被认为是对磺胺类化合物吸附的主要因素。该项研究是首次将铜功能化的三聚氰胺海绵应用于磺胺类分析物的萃取,所建立的分析方法具有富集能力强、回收率和重复性好的优点。

为探究CTFs作为磁性SPE同时萃取酸性和碱性农药的可行性。CTFs修饰磁性纳米粒子/GO复合材料被制备,其中GO的羧基和三嗪环上的-NH_2_都能够与被分析物相互作用。在最佳条件下,建立了环境水样中吡虫啉和2,4-二氯苯氧乙酸的测定方法^[85]^,两个分析物分别在0.5~500 μg/L和5.0~500 μg/L范围内呈现良好的线性关系,它们的LOD分别为0.17 μg/L和1.7 μg/L。该方法成功用于自来水样中吡虫啉和2,4-二氯苯氧乙酸的检测,结果令人满意(加标回收率≥90%)。为了检测水样中的痕量全氟化合物,Ren等^[86]^用1,4-二氰基苯和FeCl_3_·6H_2_O制备了CTF/Fe_2_O_3_微球。X射线衍射表征表明,该材料被成功合成,扫描电子显微镜、透射电子显微镜和比表面积测试法分别观察到该材料粗糙的表面、大量孔隙结构和高比表面积(864.53 m^2^/g)。基于CTF/Fe_2_O_3_的MSPE-HPLC-MS/MS方法成功用于当地池塘水质的检测。由于三嗪基团与全氟化合物之间的静电相互作用,与乙二醇改性硅胶、Oasis HLB SPE柱、C18、磁性介孔微球相比,该材料的萃取效率更高,所以该方法也更灵敏。针对环境中多溴联苯醚的检测,Liu等^[87]^以1,3,5-三(4-氨基苯基)三嗪和2,5-二甲氧基对苯二醛为原料设计合成了富含氮的亚胺基三嗪核网状CTFs, CTFs孔壁上丰富的氮/氧官能团表现出较强的电负性,使该材料和多溴联苯醚中强吸电子能力的Br产生卤键相互作用。将其作为DSPE的吸附剂与GC-MS/MS联用,建立了对多溴联苯醚高效灵敏的分析方法,并对环境水样中的目标分析物成功进行检测,结果表明该材料具有良好的稳定性和优异的吸附能力。采用简单有效的Friedel-Crafts方法合成了一种新型CTFs接枝苯基功能化SiO_2_纤维纳米球,带芴骨架的微孔CTF偶联并均匀生长在苯基功能化的SiO_2_球表面,制备了一种杂化扩展多孔骨架^[88]^。该材料用作SPME涂层萃取废水中的毒死蜱和倍硫磷农药残留,考察了离子强度、搅拌速率、样品pH、萃取温度、萃取时间等因素对萃取回收率的影响,并通过离子迁移谱法进行测定。建立的分析方法具有0.1~10 μg/L(毒死蜱)和1.0~70 μg/L(倍硫磷)的宽线性范围,0.05 μg/L(毒死蜱)和0.55 μg/L(倍硫磷)的低检出限。Wang等^[89]^通过水热法将三聚氰胺分别与1,4-哌嗪二甲醛和对苯二甲醛反应合成了两种新型CTFs(SCAU-1、SNW-1),将SCAU-1作为SPE的吸附剂,选取5种磺胺和4种四环素作为目标分析物,利用UPLC-MS/MS进行检测,建立了分析方法,并对4种实际水样(饮用水、河水、湖水和废水)进行了分析,验证了该方法的可行性。与SNW-1相比,尽管SCAU-1对磺胺的萃取性能相近,但是对四环素的萃取性能更突出。

利用类似的SiO_2_纳米球载体,本课题组^[90]^包覆了一层三嗪基聚合物,该聚合物以三聚氰胺和对苯二甲醛为原料通过席夫碱反应制备。利用环氧树脂胶水将制备的材料均匀涂覆于不锈钢丝表面,将不锈钢丝置于PEEK管中,制得IT-SPME装置。通过六通阀与HPLC连接,优化样品体积、样品流速、甲醇含量和脱附时间后建立了PAHs在线分析方法。我们还利用1,3,5-三苯基苯和三聚氯氰作为单体,通过傅克反应制备了三嗪基共价有机多孔聚合物^[91]^,其具有高度共面、大*π*共轭的三嗪骨架,相对于其他多孔材料具有更好的稳定性,而且还与芳烃类物质存在强的*π-π*相互作用。将其引入IT-SPME,通过与HPLC-DAD联用发展了灵敏、在线的分析方法(线性范围为0.013~20.0 μg/L、LOD低至0.004~0.010 μg/L、富集倍数高达1110~2763),证明了该多孔聚合物是一种可以对PAHs进行高效萃取的材料。

近年来,关于三嗪基材料的报道越来越多,三嗪基材料的发展也日趋成熟,其合成方法和功能化方法也越来越丰富。三嗪基材料作为一类优异的吸附剂材料,已经在样品前处理领域得到广泛应用。当前对于三嗪基有机骨架化合物的研究正处于初步阶段,未来对于此类材料在吸附剂萃取中的探索,将会越发受到人们的重视。

## 4 共价有机框架材料

COFs作为一类新兴的以共价键连接而成的多孔晶体有机聚合物,它由Yaghi等^[92]^在2005年基于网状拓扑学原理率先制备。COFs通常由C、H、O、N、B等轻质元素组成,可以按照连接的共价键分为硼酸酐类、硼酸酯类、席夫碱类、三嗪类、亚胺类、腙类等多种类型,发展空间巨大。COFs具有结晶度及结构稳定性好、密度低、比表面积高、结构可设计等特点,被广泛应用于吸附与分离、能源、催化等诸多领域^[93]^。基于其优异的性能,COFs也在样品前处理领域,尤其是SPME、SPE方面得到广泛关注,COFs萃取能力主要取决于材料的拓扑结构以及组成骨架的部分,因此它可以提供*π-π*、静电、亲/疏水、氢键等相互作用^[94]^,本部分对近年来COFs在SPME、MSPE中的研究进展进行了总结与概括。

TpBD-COF作为一种常见的COFs,通常利用1,3,5-三甲酰基间苯三酚(Tp)和联苯胺(BD)为单体通过水热法合成。2019年,Gao等^[95]^为快速测定水样中痕量的四溴双酚A,建立了TpBD-COF涂层的SPME-MS分析方法。该方法可在7 min内实现河水、海水、饮用水等多种水样中四溴双酚A的检测,并且还具有较宽的线性范围(0.01~10 μg/L)以及低的LOD(0.92 ng/L)与LOQ(3.1 ng/L)。然而,Gao等^[95]^提出的方法中使用的SPME基底材料玻璃纤维易碎,且物理黏附的COFs涂层易脱落。而Ma等^[96]^则利用PDA作为连接剂,将TpBD-COF作为萃取涂层接枝到不锈钢丝上,这大大提高了萃取涂层的稳定性。通过萃取涂层与目标物之间存在的*π-π*作用,该萃取纤维成功实现了多种PAHs的高效萃取。在纤维表面原位制备COFs可以改善涂层的稳定性,是一种增强萃取纤维稳定性与使用寿命的有效手段。Pang等^[97,98]^报道了借助于TiO_2_纳米管阵列将TpBD-COF接枝到钛丝纤维上,发展了一种新型的SPME纤维,用于PAEs和PAHs的萃取。通过实验也证实了,该方法发展的COFs涂层与钛丝之间存在共价键,保证了萃取纤维具有优异的萃取性能、良好的热稳定性与较长的使用寿命。Yan等^[99]^以氨基化的不锈钢丝作为载体,在室温下原位制备COFs萃取涂层,发展了一种固相微萃取纤维,用于萃取水产品中的多氯联苯,并与GC-MS/MS进行了联用,结果证明分析方法有宽的线性范围(1~1000 ng/L),高的灵敏度(LOD, 0.07~0.35 ng/L),实现了多氯联苯的有效检测。

同时,在室温下合成COFs是一种非常简便且有发展潜力的制备方式。例如,在室温条件下,三苯甲酰氯(TMC)就可以分别与对苯二胺^[100]^和1,3,5-三(4-氨基苯基)苯(TAPB)^[101]^反应生成席夫碱类型的COFs。Wang等^[101,102]^将这两种COFs作为萃取涂层,建立了两种顶空SPME-GC-MS的分析方法,并借此分别检测了空气中的苯系物与水样中的PAHs,实验结果也证明了由于*π-π*效应等,在室温下制备的COFs涂层也对目标分析物表现出了良好的萃取效果。

近年来,Zhao的课题组^[103,104,105,106]^也发展了一些新型的COFs,并将其应用于SPME领域。例如,他们合成了一种席夫碱类型的TpPa-1-COF(Pa-1,对苯二胺)用作SPME萃取涂层,分别萃取了水样中的多溴联苯醚和人工合成麝香^[102,103]^,通过与GC-MS联用建立的分析方法,实现了对痕量目标分析物的有效检测,扩展了COFs在SPME领域中的应用。此外,他们小组还在室温下合成了球形TPB-DMTP-COF(TPB: 1,3,5-三(4-氨苯基)苯;DMTP: 2,5-二甲氧基苯-1,4-二甲醛)^[104]^。通过表征发现,TPB-DMTP-COF不仅具有良好的结晶度还具备高达1560 m^2^/g的比表面积。将TPB-DMTP-COF用作SPME纤维萃取涂层,通过与GC-MS/MS联用,建立了一种针对地下水中苯酚的分析方法。基于该COFs与苯酚之间的强作用力,该方法实现了超低的LOD(0.015 ng/L)以及高达4265的富集倍数。

在SPME领域中利用创新性方法发展新的COFs作为萃取涂层,往往是一种大胆的尝试。Yamini课题组^[107]^尝试以三聚氯腈和对羟基苯甲醛为单体通过简单的取代反应合成了2,4,6-三(4-甲酰基苯氧基)-1,3,5-三嗪,之后又以其为单体与肼在室温下生成了一种亚氨键连接的共价三嗪有机骨架材料。利用与目标物之间的*π-π*、氢键等作用力,该COFs作为SPME萃取涂层成功实现了对废水中几种氯酚的有效萃取。此外他们也尝试通过掺杂GO制备了一种纳米碳材料复合的COFs材料^[108]^。Tian等^[109]^则在SPME中引入了一种卟啉结构的TFPA-TAPP-COF(TFPA:三(4-甲酰基苯基)胺;TAPP:四(4-氨基苯基)卟啉)涂层,并通过层层自组装技术将其生长在不锈钢丝表面,制备了一种新型的萃取纤维。通过与GC-MS联用建立分析方法,实现对水样中PAHs的分析检测(线性范围为0.1~50 μg/L, LOD为0.006~0.024 μg/L),证实了该COFs具有良好的萃取性能。

Ji课题组^[110]^将现代有机合成技术应用于COFs材料的快速微波制备,首次基于不可逆反应,以2,3,5,6-四氟-4-吡啶二甲腈和2,3,6,7,10,11-六羟基苯并菲为原料,在30 min内合成了二氧六环骨架COFs(TH-COFs)。利用微波辅助合成的TH-COFs具有网状形貌和超高比表面积(1254 m^2^/g)。将TH-COFs用于固相微萃取涂层后,详细优化了萃取条件,建立了水样中8种全氟化合物的TH-COFs-SPME-UPLC-MS/MS检测方法,LODs≤0.0045 ng/L。针对该类分析物,该小组也发展了一种氟功能化COFs的萃取涂层,借助于氟-氟亲和作用,实现了乳制品中全氟化合物的良好萃取^[111]^。以上工作证明了设计和合成功能化COFs材料可以提高对特定目标分析物的萃取选择性,这对COFs在SPME领域的发展具有很好的启发性。

COFs复合其他材料是进一步改善相互之间萃取性能的一种行之有效的手段。例如,Tian等^[112]^制备的TpBD-COFs/GO复合材料,萃取能力分别是TpBD-COFs和GO单个材料萃取能力的2.2倍和4.7倍。最近,一种新型的多刺枝状石墨氮化碳(g-C_3_N_4_)-TpBD-COFs的复合材料通过溶胶-凝胶法被包覆在不锈钢丝上,并应用于环境中8种PAHs的SPME研究中^[113]^。g-C_3_N_4_本身具有共轭电子结构,有利于对芳香族化合物(如PAHs)进行萃取,然而受困于本身的低比表面积以及容易团聚的缺陷,大大限制了它的萃取性能。而利用TpBD-COFs对其进行改性,可以充分弥补这些缺点,复合材料所拥有的特殊形貌也大大提高了比表面积。同时研究发现,这种多刺枝状结构只有当g-C_3_N_4_与组成COFs单体的质量比合适时才会出现。

COFs也常常作为MSPE材料用于样品前处理中。Wang等^[114]^先在磁性Fe_3_O_4_上包覆一层COFs,再通过点击反应利用乙二硫醇对COFs进行功能化,制备了一种海胆状的Fe_3_O_4_@COF-S-SH萃取材料。该萃取材料对无机汞(IHg)、甲基汞(MeHg)和乙基汞(EtHg)分别表现出了高达571、559与564 mg/g的良好吸附能力,通过建立的MSPE-HPLC-电感耦合等离子体质谱(ICP-MS)分析方法实现了对汞的有效检测。在MSPE领域中,混合型萃取剂能够实现对多类别分析物的有效萃取,具有良好的应用潜力。Hu等^[115]^通过在Fe_3_O_4_表面生成带有羧基的COFs,不仅借助于COFs本身的芳环促进了疏水和*π-π*共轭作用,独特的孔隙能够与目标物进行充分接触,而且引入的羧基更是可以参与弱阳离子的交换作用,并借助“同步萃取-逐步脱附”的策略,成功地实现了对PAHs、四环素以及三苯甲烷染料的有效萃取。Lin等^[116]^另辟蹊径,利用磁性碳纳米管作为新型载体,将氟化COFs作为萃取涂层引入。由于氟基团的引入,萃取材料的疏水性得到了加强。所构建的MSPE-GC-MS分析方法具有极低的LOD(0.0045~0.018 ng/L)与良好的线性范围(0.01~500 ng/L),可实现对超痕量多溴联苯醚的有效萃取。

除此之外,COFs也被负载在滤纸表面作为一种纸基的萃取方法用于富集环境样品中的四溴双酚A^[117]^,这项研究也拓展了COFs在不同样品前处理形式上的应用。综上,这些研究工作对COFs在SPME、MSPE等样品前处理领域中的应用奠定了良好基础,并对其在该领域的进一步发展具有促进作用。

## 5 分子印迹聚合物

MIPs的制备方法主要包括本体聚合法、沉淀聚合法、微乳液聚合法、悬浮聚合法、原位聚合法、多步溶胀聚合法等^[118]^。它与模板分子在形状、尺寸和功能基团方面是互补的。这些独有的特征使MIPs能够特异性识别和选择性吸附目标分析物,这也使它在萃取领域发挥着重要作用。本部分对MIPs萃取材料在搅拌棒萃取、MSPE、PTSPE、SPME等领域的应用进行了总结和概括。

分子印迹整体柱在SPE方面占有重要地位。对于手性污染物的准确分析一直是一个具有挑战性且亟须解决的问题。Liu等^[119]^将手性模板与手性功能单体通过点击反应结合,制备了一系列手性MIPs搅拌棒,结合HPLC-DAD检测方法,建立分析方法用于富集和检测环境水样(湖水,河水)中萘普生对映体含量,可以获得满意的回收率(83.98%~118.88%)。百草枯作为高毒性禁用农药,在环境水样中对其进行高灵敏检测,对于食品安全和环境安全具有重要意义。Yao等^[120]^采用百草枯为模板,通过溶胶-凝胶技术,将印迹聚合物固定在端羟基聚二甲基硅烷玻璃搅拌棒表面,得到MIPs涂层搅拌棒。对于环境水样中的阳离子百草枯具有特异性识别能力和优良的吸附能力。将搅拌棒萃取和HPLC结合,检出限低至8.2 ng/L,线性范围为100~10000 ng/L。该方法对于水样中痕量农药的检测具备较高的灵敏度。

Tian等^[121]^以17*β*-雌二醇为模板分子,以多巴胺为功能单体,在羧基功能化的Fe_3_O_4_纳米颗粒载体表面,制备MIPs涂层,将该复合材料作为MSPE的萃取剂,借助于HPLC测定,对环境水体中的微量17*β*-雌二醇进行高灵敏度分析,获得了令人满意的LOD(0.008 μg/L)。萃取材料在10次测试之后,吸附效率仍可达到原来的94.8%。Deng等^[122]^结合表面分子印迹、多模板和磁分离的优点发展了一种MIPs,通过MSPE和HPLC-UV,建立了针对地表水、地下水和生活污水中邻苯二甲酸酯类污染物(邻苯二甲酸二甲酯、邻苯二甲酸二乙酯、邻苯二甲酸二丁酯)的分析方法。

Abu-Alsoud等^[123]^将玻璃片在甲苯中用3-(三甲氧基硅基)甲基丙烯酸丙酯衍生后,将预聚合溶液覆盖到衍生化的玻璃片上,然后用石英盖玻片轻轻覆盖,暴露于紫外光下进行光聚合,制备得到MIPs膜。通过膜固相萃取结合液相色谱分析检测,建立了适用于测定海水中具有多极性的苯酚、烷基酚、氯苯酚等微量酚类化合物含量的分析方法。

区别于传统SPE技术,PTSPE具有装置简便、操作简单、耗时短、环境友好等优势。它是采用移液枪头作为SPE装置,只需要消耗少量吸附剂和有机溶剂即可完成高效样品前处理。针对基质复杂的样品,通过PTSPE技术,仅仅需要少量的样品体积即可对目标分析物实现快速、准确地萃取和富集,进而提高分析方法的灵敏度和可信度。Hashemi等^[124]^以甲基红为模板分子,甲基丙烯酸为功能单体,乙二醇二甲基丙烯酸酯为交联剂,采用本体聚合方法制备了甲基红的MIPs。采用一次一变量和响应面法研究了萃取条件。在最佳条件下,用UV对海水样品中的甲基红进行了分析,LOD为0.5 μg/L,回收率为84.0%~98.0%。采用相同的制备方法,他们研制了另一种MIPs,从海水中萃取多种染料包括孔雀石绿、罗丹明B、甲基橙和酸性红18^[125]^。该课题组将MIPs与非印迹聚合物(NIPs)进行了萃取性能对比,结果表明MIPs具有更好的吸附容量和选择性,因此将PTSPE-HPLC联用,建立分析方法,具有低的LOD(0.083~0.17 μg/L)、宽的线性范围(0.5~250.0 μg/L)和令人满意的加标回收率(76.1%~97.3%)。同年,Hashemi等^[126]^将ZnO纳米粒子引入到聚合物的制备中,得到MIPs/ZnO复合材料,ZnO纳米粒子有助于提高萃取剂的稳定性和耐用性,并且有助于提高材料的比表面积,进而提升萃取性能。通过PTSPE结合UV,建立了回收率(93.1%~99.1%)良好的分析方法,应用于海水中尼古丁的分析检测。次年,该课题组采用环丙沙星作为模板分子合成了一种用于从海水中富集目标分析物的萃取剂,且表现出良好的萃取效果^[127]^。

与传统的MIPs制备方法不同,Teixeira等^[128]^采用聚(1-乙烯基咪唑-三甲基丙烷三甲基丙烯酸酯)作为一种新的MIPs,对水样中阿维菌素和米贝霉素进行了萃取。将富集之后的抗生素经过HPLC进行分析检测,使得线性范围为25~750 ng/mL。Cao等^[129]^分别使用2,4-二氯苯氧乙酸和4-乙烯基吡啶作为模板和功能单体制备了分子印迹材料。通过混合MIP和高密度聚乙烯,在150 ℃条件下进行加热,制备了萃取装置。在HPLC测定前,利用PTSPE进行富集,可以得到良好的灵敏度和回收率分别为0.002 mg/L和114.2%~122.1%,并成功应用于实际水样中2,4-二氯苯氧乙酸的分析检测。近年来,随着ILs在萃取领域的不断兴起,通过选择其作为功能性单体,可以进一步改善MIPs的缺点。它可以涉及多重萃取机理,如离子交换、*π-π*堆积和静电作用,使得它在环境水样中对目标分析物具有更好的吸附性能。它还可以作为溶剂或致孔剂,改善分子印迹吸附剂的性能。

分子印迹材料在SPME中也被广泛采用,Wang等^[130]^设计了一种以MIPs为纤维涂层的纤维阵列萃取法,可以用于同时富集三类环境雌激素。与单一纤维相比,纤维阵列具备更高的吸附容量,可有效提高分析方法的灵敏度和准确性。经过HPLC分析得到的双酚和对羟基苯甲酸酯的检出限为0.003 μg/L, PAEs检出限为0.016 μg/L,回收率为80.8%~114.1%。磷酸三苯酯作为一种有机磷阻燃剂,是一种对人体健康有害的新型污染物,Jian等^[131]^以氧化石墨烯为载体制备MIPs,提高了材料的比表面积、亲水性和萃取效率。借助气相色谱-火焰光度检测(GC-FPD)建立了针对自来水、河水和湖水中痕量磷酸三苯酯污染物的分析方法,获得良好的检出限和线性范围,分别为0.0001 ng/mL和0.0007~124 ng/mL。Fang等^[132]^在毛细管内原位制备了磁性MIPs整体萃取柱,由于分子印迹和磁性增强的协同作用,实现了对衍生分析物的选择性和高效萃取,同时去除了多余的衍生试剂,可以直接分析水样中的微量醛。所建立的IT-SPME联用毛细管电泳分析方法,对地表(饮用)水的检测灵敏度较高,LOD为0.0032~0.0049 mg/L。

MIPs是一类优异的高选择性萃取材料,它的识别位点可以特异性地吸附目标分子,在样品前处理方面已得到广泛应用。新型的MIPs及其应用的不断改进将会推动样品前处理技术的不断发展。

## 6 金属有机框架材料

MOFs是由金属离子与有机多齿配体通过物理或化学方法配位形成的一类有机-无机杂化多孔晶体材料。这类材料不仅具有较大的比表面积和较多的孔径分布,还具有孔道规则和孔径尺寸可调等优点^[133]^,使其能够被广泛应用在催化、气体储存、吸附与分离、载药传递和光学器件等领域。近年来,MOFs被引入样品前处理领域,并发挥着越来越重要的作用。

近年来,MOFs在SPE领域的研究得到较大发展,特别是在环境监测方面。Ma等^[134]^于2020年以四氟对苯二甲酸为配体,Zn(Ⅱ)为中心金属离子,成功制备了一种新型氟化多孔膜作为SPE吸附剂。配体上的氟基团通过氟-氟强亲和力,对全氟辛烷磺酸表现出优异吸附能力,并且具有良好的稳定性和重复性。结合GC-MS检测,建立相关的分析方法并应用于环境水样中全氟辛烷磺酸的检测(最大吸附量为419.8 mg/g, LOD为2.6 ng/L)。Boontongto等^[135]^将MOFs和磁铁矿结合制备了一种磁性MIL-53(Al)-NH_2_,并提出了一种简单快速的涡流辅助分散MSPE方法,从水样中萃取了美国环保部优先控制的10种酚类,随后通过HPLC-DAD检测。所开发的方法能够在10 s内快速富集目标分析物,且效率高,重复性好(RSD<12%)。Li等^[136]^通过简单的溶剂热法一步合成了一种新型MOF-1210(Zr/Cu)修饰的磁性纳米粒子,用于二苯甲酮的磁固相萃取, MOF-1210(Zr/Cu)与二苯甲酮之间的氢键作用和配位作用是主要的萃取机理。基于该磁固相萃取的分析方法得到了满意的结果(富集倍数为91~122倍,线性范围为0.1~300.0 ng/mL),成功应用于土壤样品中二苯甲酮的萃取和检测。Wang等^[137]^在最佳热解温度下碳化Zn/Co-MOF-5制得磁性多孔碳,作为MSPE吸附剂应用于4种氨基甲酸酯的萃取。与HPLC-MS联用,建立了高灵敏的分析方法,得到的LOD低至0.0006 ng/mL,并成功应用于水样分析。

ZIF-8作为一种常见的MOFs材料,已被广泛应用于吸附、分离等领域,为了提高萃取效率,正丁胺分子被引入到ZIF-8骨架结构中^[138]^,制备了NH_2_-ZIF-8,有利于调整表面性质,提高对目标分析物的萃取选择性。在最佳条件下,通过SPE结合HPLC-UV检测,对环境污水和土壤中的2-巯基苯并噻唑、2-巯基苯并唑唑和2-巯基-6-硝基苯并噻唑进行了分析检测。近年来,SPE柱应用广泛,MOFs由于颗粒直径较小,将其装填进SPE柱,会造成高背压,导致结构坍塌,影响材料的稳定性和萃取性能。G作为一种优良的吸附材料,具备良好的疏水性,相比G, GA是一种更有吸引力的材料,其3D网络提供了大的表面积和孔径。Zhou等^[139]^以GA作为载体,利用水热合成法制备了MIL-101(Cr)@GA作为SPE柱萃取材料,并用于富集环境水样中5种非甾体类消炎药(NSAIDs),并获得了良好的灵敏度,检出限为0.006~0.012 ng/mL,相对标准偏差为0.6%~8.4%。Jiang等^[140]^直接将MIL-101(Cr)和壳聚糖直接嵌入到三聚氰胺海绵材料的骨架上,作为萃取柱吸附材料使用。壳聚糖不仅在制备功能化海绵的过程中充当黏合剂,而且具备一定的吸附能力和许多吸附位点。壳聚糖具有优异的亲水性,这使得萃取柱可以用于水样检测。借助涡流辅助SPE和LC-MS,完成了水样中微量三嗪除草剂的检测,检出限为0.014~0.045 ng/mL。

Zr-MOFs的机械稳定性和化学稳定性低,限制了它们在水溶液中的应用。通过结合其他客体,MOFs的吸附性能和水稳定性将进一步提高。这类复合材料的开发是当下热门话题。Lu等^[141]^采用咪唑基ILs作为客体分子功能化Zr-MOFs,然后用羟基、羧基、氨基或苄基官能团对ILs的阳离子进行修饰,可以提高对水样中磺胺类抗生素的萃取效率,且ILs@Zr-MOFs在水中具备良好的稳定性。该复合材料基于大比表面积、静电、氢键相互作用,通过DSPE对环境水样中磺胺类抗生素表现出良好的萃取效率,富集因子为270~300。为了节省萃取时间,他们在前期工作的基础上开发了一种新型的磁性复合材料,并用于MSPE。以PDA功能化的Fe_3_O_4_为核心^[142]^,在其表面经过逐层修饰得到带正电荷的Fe_3_O_4_@Zr-MOFs,将羧基功能化的ILs(IL-COOH)包裹进Fe_3_O_4_@Zr-MOFs的孔道中,提高了选择性和萃取效率,成功获得了新型磁性纳米复合材料(IL-COOH/Fe_3_O_4_@Zr-MOFs),其中IL-COOH/Fe_3_O_4_@UiO-67-bpydc被用来检测水样中的氟喹诺酮类抗生素,富集因子和检出限分别为450~500 μg/L和0.01~0.02 μg/L。Niu等^[143]^采用原位生长法制备了一种用凹凸棒石(ATP)改性的磁性MOF复合材料ATP@Fe_3_O_4_@MIL-100(Fe)。利用MSPE技术和HPLC,同时测定了环境水样中7种苯甲酰脲类杀虫剂,LOD和线性范围分别为0.75~1.5 μg/L和2.5~500 μg/L。

MOFs材料的疏水性限制了它们在水样中的分散性,为了将MOFs更加广泛地应用于样品前处理中,Tan等^[144]^将DSPE和液相微萃取结合,开发了一种新型溶剂负载DSPE技术。他们将有机溶剂二氯甲烷负载到用-NH_2_功能化的MIL-101(Cr)孔道中,二氯甲烷可以促进分析物的液相微萃取。借助UPLC-MS分析稻田水体中5种氯苯氧基酸除草剂含量,得到较低的检出限(2.66~19.7 ng/L)。未来,可以设计自动采样装置,为样品前处理技术的新发展提供思路。

其次,MOFs在PTSPE方面也有较好的发展。2018年,Zr-MOF-NH_2_被制备并作为吸附剂,对水样中卡马西平进行富集,进而通过HPLC进行分析检测,该方法的LOD低至0.04 μg/L^[145]^。高的萃取性能可能是因为-NH_2_和-OH之间的氢键作用。Kahkha等^[146]^使用快速超声辅助合成法制备了Co-MOF,将该吸附剂填充进移液枪头尖端作为萃取装置,用HPLC有效分析饮用水中的双酚A, LOQ低至0.07 μg/L,线性范围为0.3~300.0 μg/L^[147]^。该吸附剂还用于海水中有机染料(孔雀石绿、罗丹明B、甲基橙、酸性红18)的富集,对于基质复杂的海水样品,LOD为0.09~0.38 μg/L^[146]^。2019年,他们采用微波辅助反胶束法快速制备了比表面积高达2324 m^2^/g的Ta-MOF,并将其应用于PTSPE,利用HPLC得到了令人满意的检测范围(2.3~3000.0 μg/L)^[148]^。Amini等^[149]^采用静电纺丝法制备PAN/MIL-53(Fe)纳米纤维并将其用于PTSPE。结合HPLC检测方法对废水中的痕量苯二氮卓类药物进行分析,加标回收率为92.0%~100.0%,并且仅需要2 min即可完成对样品的前处理,大大节省了复杂基质样品的前处理时间。NH_2_-MIL-68@COF作为PTSPE的吸附剂,被用于环境水样中6种磺胺类药物的快速富集。通过HPLC分析,线性范围宽至10.0~2000.0 μg/L。Nurerk等^[150]^采用水热合成法制备了MIL-101,用于移液枪头固相萃取,并通过GC-MS/MS对不同水样(矿物、河流、废水、游泳池水、海水)中的紫外线吸收剂进行分析。

ZIF-8是一种发展比较成熟的、应用广泛的沸石咪唑酯骨架结构的MOFs材料,将其制备为SPME涂层,应用于PAHs及一些酯类个人护理产品(水杨酸三甲环己酯、硬脂酸2-乙基己酯、芳酸甲酯、4-(二甲氨基)苯甲酸-2-乙基己酯、4-甲氧基肉桂酸2-乙基己酯)的萃取^[151]^。该工作首先利用气相沉积法制备了ZIF-8种子层,利用溶剂热法控制萃取涂层生长到3 μm。这项工作创新性地利用两种方法制备了MOFs萃取涂层,原位生长且厚度均匀、可控,稳定性好,萃取性能能够媲美商品化涂层。Suwannakot等^[152]^则开发了借助于MS直接快速检测和定量分析环境水样中的全氟辛酸4种MOFs涂层(ZIF-8、UiO-66、MIL88-A和Tb_2_(BDC)_3_)的探针,这些探针可以在无需样品处理的情况下在5 min之内实现对实际样品的定量分析。这为现场实时、准确地检测环境污染物提供了一种非常好的思路。

MOFs的化学结构丰富多样,并且可以根据需要进行目标设计。MOFs作为一类优良的吸附材料,具有丰富的孔隙,孔径大小可调节,这为分析物的进入和转移提供了大量通道。其次,大的比表面积可以提供足够多的吸附位点,获得高的萃取容量。MOFs的结构可调,可以根据需要修饰功能基团,提高萃取性能。它还表现出优异的化学稳定性。这些优点使得它在萃取领域越来越被人们所关注,虽然已报道的MOFs已经有许多种类,但仍有巨大的发展空间。

## 7 结论

本文对近年来几类新型的样品前处理材料研究进展进行了详细总结,主要包括近年来研究比较热的G和CNTs两大类碳纳米材料、气凝胶、三嗪基材料、MIPs、COFs、MOFs等。这几类代表性的萃取材料虽然不能全部涵盖样品前处理的所有进展,但是也可以为本领域及相关研究人员提供一些有价值的参考。虽然近年来样品前处理获得了很好的发展,出现了多种类型的新型样品前处理技术以及种类丰富的萃取材料,但是仍然存在一些有待改善的方面,比如:在线样品前处理和自动化分析检测的技术较少,材料易存在残留效应,材料的耐热性和耐溶剂性不够理想,材料的制备过程步骤多,条件要求高,材料不够环保绿色,专一选择性的样品前处理材料较少,样品基体对于材料萃取性能影响大等。

针对这些问题,我们展望样品前处理未来的几个发展趋势:1)发展高效的样品前处理材料,具备高富集能力、高选择性、优异的热稳定性和化学稳定性;2)开发绿色的样品前处理材料,发展环保的材料合成方法,探索绿色的样品前处理方法;3)开发快速的样品前处理方法,能够在数分钟甚至数秒内完成分析物快速富集;4)发展在线样品前处理技术,以及自动化的分析检测方法;5)实时分析、原位检测是未来一个重要的发展方向,在环境分析、生物检测等领域具有重要的应用;6)将现代合成技术应用于萃取材料的合成,制备目标萃取材料;7)萃取材料的可控制备方法将会越来越被重视,如原位生长或原位制备萃取涂层已被应用;8)将其他领域的高性能材料引入样品前处理领域,实现交叉领域的融合;9)有机-无机杂化萃取材料能够兼具二者优点而相互弥补缺点,是一个值得深入探索的方向,纳米材料掺杂的萃取材料也是一个重要的发展方向;10)现有样品前处理技术虽然比较丰富,但是发展创新性的样品前处理技术仍是至关重要的。
